# A retrospective study of surgical treatment and outcome among women with adnexal torsion in eastern Taiwan from 2010 to 2015

**DOI:** 10.7717/peerj.5995

**Published:** 2018-12-04

**Authors:** Ci Huang, Mun-Kun Hong, Tang-Yuan Chu, Dah-Ching Ding

**Affiliations:** 1 Department of Obstetrics and Gynecology, Hualien Tzu Chi Hospital, Buddhist Tzu Chi Medical Foundation, Tzu Chi University, Hualien, Taiwan, Taiwan; 2 Institute of Medical Sciences, Tzu Chi University, Hualien, Tawian, Taiwan

**Keywords:** Torsion, Laparoscopy, Adnexa, Cystectomy, Ovary

## Abstract

**Background:**

Adnexal torsion is a gynecologic emergency that requires surgical treatment. In this study, we reviewed the surgical outcomes of women with adnexal torsion in eastern Taiwan (Hualien county, area 4,629 km^2^, 330,000 residents).

**Methods:**

This retrospective study included 42 women diagnosed with surgically-proven adnexal torsion from January 1, 2010, to September 31, 2015. We compared the symptoms, objective findings, and surgical outcomes of patients who underwent laparotomy or laparoscopy.

**Results:**

The laparoscopy and laparotomy groups included 27 and 15 patients, respectively. The most common symptom and sign was abdominal pain, followed by nausea and vomiting. In all patients, an adnexal tumor was detected through ultrasound. The median and range of time from admission to surgery was 1.5 (1–11.5) and 1.0 (1–11) hours in the laparotomy and laparoscopy groups, respectively. Compared with those undergoing laparotomy, the smaller tumor size [7 (4.2–10) vs. 10 (7–17) cm] and shorter hospital stay [4 (2–8) vs. 6 (3–9) days] in patients undergoing laparoscopy were significantly noted, respectively (*P* < 0.01). No differences were observed in age, operative time, and blood loss between both groups. The surgeries performed were mostly detorsion with cystectomy and adnexectomy. The most common pathology was a simple ovarian cyst, followed by teratoma. Regarding the surgical types, older age is the only risk factor for radical surgery.

**Discussion:**

Acute onset of abdominal pain with a presenting ovarian tumor is the most common feature of adnexal torsion. Laparoscopic surgical group showed a small tumor size and a short ER hospital stay than laparotomy. Older age is the risk factor for radical surgery.

## Introduction

Adnexal torsion is a gynecologic emergency that requires surgical treatment (Huang & Wang, 2011; Huang, Hong & Ding, 2017). It is defined as twisting of the ovary, fallopian tube, or adnexal mass, inducing adnexal torsion. Partial or complete rotation of the ovarian vascular pedicle obstructs venous outflow and arterial inflow (Chang, Bhatt & Dogra, 2008). Both benign and malignant lesions of the ovary may be the leading causes of adnexal torsion.

Surgical intervention is the gold standard for diagnosis and treatment of adnexal torsion. Conventionally, a twisted ovary or adnexa is excised completely (Houry & Abbott, 2001). However, adnexa-sparing surgery has emerged as an alternative (Ding & Chen, 2005; Spinelli et al., 2013; Nair, Joy & Nayar, 2014; Ding & Chang, 2016). Conservative surgery such as detorsion with cystectomy or cyst aspiration is preferred to preserve adnexal function. A previous study revealed that 51.4% of patients presenting to the emergency room were diagnosed with adnexal torsion (Lo et al., 2008).

Therefore, this study investigated the clinical characteristics of women with adnexal torsion in eastern Taiwan and compared the surgical outcomes of laparotomy and laparoscopy. We also calculate the risk factor for radical surgery.

## Methods

This retrospective study analyzed the discharge data of women diagnosed with surgically proven adnexal torsion at Hualien Tzu Chi Hospital from January 1, 2010, to September 31, 2015. This study was approved by the Research Ethics Committee of Hualien Tzu Chi Hospital (IRB107-20-B). The Ethics Committee waived the need for informed consent from participants of this study.

All patients diagnosed with adnexal torsion had records of the International Classification of Diseases, Ninth Revision, Clinical Modification (ICD-9-CM) code 620.5 in their discharge notes. A total of 45 women were enrolled into our study, and 42 were diagnosed with adnexal torsion after surgery. Three patients did not receive surgical intervention because they refused surgical intervention, were lost to follow-up, or opted to receive medical treatment. Information on clinical characteristics, including age, medical history, operative time, and surgical methods, was obtained from patients’ electronic medical records. Abdominal pain was defined as diffuse pain in the lower abdomen. In addition, surgical findings and pathological reports were obtained.

The surgical routes were laparotomy and laparoscopy. The surgical methods for the management of adnexal torsion were cystectomy, adnexectomy, and detorsion.

Statistical analyses were performed using SPSS version 25 (IBM, New York, NY, USA). All continuous variables are presented as median (range). All categorical variables are presented as numbers (percentage). Mann–Whitney *U* test was used to compare the average of variables between two groups to determine the association between two continuous variables. Fisher’s exact test was used to determine the difference between two categorical variables. Logistic regression was used to determine the odds ratio in the radical surgery group compared to the conservative surgery group. A *P* value of <0.05 was considered statistically significant.

## Results

A total of 42 patients were surgically diagnosed with adnexal torsion during the study period. Table 1 illustrates the symptoms and signs of patients with adnexal torsion. The laparoscopy and laparotomy groups included 27 and 15 patients, respectively. In both groups, the most common symptom was lower abdominal pain (95.2%), followed by nausea and vomiting experienced by three and five patients in the laparoscopy and laparotomy groups, respectively. Regarding other symptoms, one patients in the laparoscopy group developed urinary symptoms, and two in the laparotomy group developed diarrhea. On examination, adnexal masses (100%) were noted in all patients. Four patients in the laparoscopy group and two in the laparotomy group had leukocytosis. One patient in the laparotomy group had fever, and one in each group had peritoneal signs. In our series, adnexal torsion was suspected in 64.4% of patients.

**Table 1:** Preoperative symptoms and signs associated with adnexal torsion (*n* = 42).

	Laparoscopy (*n* = 27)	Laparotomy (*n* = 15)	*P*-value
Ovarian or pelvic mass	27	15	
Pelvic pain	26	14	0.59
Nausea and vomiting	5	3	0.60
Peritoneal sign	1	1	059
WBC > 12,000	4	2	0.63
Fever	0	1	0.35
Urinary symptoms	1	0	0.64
Diarrhea	0	2	0.15

**Notes.**

*P*-value: Fisher’s exact test.

The median time (range) from symptom onset to seeking medical help was 12 (0–36) and 24 (3–96) hours in the laparoscopy and laparotomy groups, respectively (*p* = 0.03, Table 2). The median time (range) from admission to surgery was 1.5 (1-11.5) and 1.0 (1–11) hours in the laparoscopy and laparotomy groups, respectively. The median time (range) from gynecologic evaluation to surgery was 2.0 (1–11) and 2.0 (1–11) hours in the laparoscopy and laparotomy groups, respectively.

**Table 2:** Time interval between variables.

	Laparoscopy (*n* = 27) Median (range)	Laparotomy (*n* = 15) Median (range)	*P*-value
From symptom onset to ED admission (hr)	12 (0–36)	24 (3–96)	0.03^*^
From admission to surgery (hr)	1.5 (1–11.5)	1.0 (1–11)	0.81
From gynecologic evaluation to surgery (hr)	2.0 (1–11)	2.0 (1–11)	0.85

**Notes.**

*P*-value: Mann–Whitney *U* test.

ED, emergency department.

* *P*-value < 0.05 was considered statistically significant after test.

Table 3 presents the pathological findings of adnexal torsion. The most frequent pathology was a simple cyst (*n* = 17, 13 and 4 in the laparoscopy and laparotomy groups, respectively), followed by mature cystic teratoma (*n* = 10, 6 and 4 in the laparoscopy and laparotomy groups, respectively). The remaining pathologies were endometrioma, fibroma, complex ovarian cyst and ovarian necrosis.

**Table 3:** Pathological diagnosis of the adnexal tumor.

	Laparoscopy (*n* = 27)	Laparotomy (*n* = 15)	*P*-value
Simple cyst	13	4	0.15
Mature cystic teratoma	6	4	0.51
Endometrioma	4	1	0.40
Fibroma or fibrothecoma	0	4	0.01^*^
Necrosis of ovary	1	0	0.64
Complex cyst	3	2	0.59

**Notes.**

*P*-value: Fisher’s exact test.

* *P*-value < 0.05 was considered statistically significant after test.

Table 4 provides a comparison of the surgical characteristics of patients who underwent laparotomy or laparoscopy. The median (range) age of patients was 31 (13–76) and 41 (12–82) years in the laparoscopy and laparotomy groups, respectively. The median (range) operative time was 64 (20–200) and 70 (45–168) minutes in the laparoscopic and laparotomy groups, respectively ( *P* = 0.50). The median (range) blood loss was 50 (50–500) ml and 50 (50–400) ml in the laparoscopy and laparotomy groups, respectively (*P* = 0.30). The median (range) tumor size was 7 (4.2–10) and 10 (7–17) cm in the laparoscopy and laparotomy groups, respectively (*P* < 0.01). The median hospital stay was significantly shorter in the laparoscopy group than in the laparotomy group (4.0 vs. 6.0 days, *P* < 0.01). The total number of cases managed by cystectomy was 12, in which 10 and 2 cases were managed by laparoscopy and laparotomy, respectively (*P* = 0.09). The total number of cases managed by adnexectomy was 27, in which 15 and 12 cases were managed by laparoscopy and laparotomy, respectively (*P* = 0.10). Other two surgeries were drainage and fixation by laparotomy.

**Table 4:** Surgical characteristics in patients with adnexal torsion underwent laparoscopy and laparotomy.

	Laparoscopy (*n* = 27) Median (range)	Laparotomy (*n* = 15) Median (range)	*P*-value
Age (year)	31 (13–76)	41 (12–82)	0.26
Surgical time (min)	64 (20–200)	70 (45–168)	0.50
Blood loss (mL)	50 (50–500)	50 (50–400)	0.30
Tumor size (cm)	7 (4.2–10)	10 (7–17)	<0.01^*^
Hospital stay (day)	4 (2–8)	6 (3–9)	<0.01^*^
Surgery^a^*n* (%)			
Detorsion and drainage	1 (3.7%)	0 (0%)	0.64
Detorsion and cystectomy	10 (37.0%)	2 (13.3%)	0.09
Detorsion and fixation	0 (0%)	1 (6.6%)	0.35
Ovarian or adnexal resection	15 (55.6%)	12 (80.0%)	0.10

**Notes.**

*P*-value: Mann–Whitney *U* test.

a *P*-value: Fisher’s exact test.

* *P*-value < 0.05 was considered statistically significant after test.

Table 5 compared the basic characteristics, surgical, and pathological parameters between conservative and radical surgery groups. We found the age was significant different between the both groups (*P* = 0.001). Older age was noted in radical surgery group. The other parameters was no difference between both groups.

**Table 5:** Surgical characteristics in patients with ovarian torsion underwent radical or conservative surgery.

	Radical surgery (*n* = 27) Median (range)	Conservative surgery (*n* = 15) Median (range)	*P*-value
Age (year)	42 (12–82)	24 (13–45)	<0.01*
Surgical time (min)	70 (29–190)	60 (30–200)	0.66
Blood loss (mL)	50 (50–500)	50 (50–500)	0.66
Tumor size (cm)	8.5 (4.2–17)	7.0 (4.6–12)	0.11
Hospital stay (day)	5 (2–9)	4 (2–7)	0.11
Time interval			
From symptom onset to ED admission (hr)	12 (0–48)	12 (1–96)	0.76
From admission to surgery (hr)	1.5 (1–11)	1 (1–11.5)	0.84
From gynecologic evaluation to surgery (hr)	2 (1–11)	2 (1–11)	0.97
Op type [*n* (%)]			0.1
Laparoscopy	27 (55.6%)	12 (80%)	
Laparotomy	12 (44.4%)	3 (20%)	
Tumor type (*n*)			
Simple cyst	9 (33.3%)	8 (53.3%)	0.17
Mature cystic teratoma	6 (22.2%)	4 (26.7%)	0.51
Endometrioma	4 (14.8%)	1 (6.7%)	0.4
Fibroma or fibrothecoma	4 (14.8%)	0 (0%)	0.15
Necrosis of ovary	1 (3.7%)	0 (0%)	0.64
Symptoms and signs (*n*)			
Ovarian or pelvic mass	27 (100%)	15 (100%)	1
Pelvic pain	25 (92.6%)	15 (100%)	0.5
Nausea and vomiting	3 (11.1%)	5 (33.3%)	0.09
Peritoneal sign	2 (7.4%)	0 (0%)	0.4
WBC > 12,000	5 (18.5%)	1 (6.7%)	0.53
Fever	1 (3.7%)	0 (0%)	0.39
Urinary symptoms	0 (0%)	1 (6.7%)	0.35
Diarrhea	1 (3.7%)	1 (6.7%)	1

Table 6 calculated the odds ratio between between conservative and radical surgery groups. We found older age is the risk factor for radical surgery [adjusted odds ratio (95% CI) =1.14 (1.04–1.24), *p* = 0.004]. Patients with nausea or vomiting revealed a low risk for radical surgery (adjusted odds ratio (95% CI) =0.02 (0.00–0.97), *p* = 0.048).

**Table 6:** Factors associated with receiving oophorectomy (*n* = 42).

	Crude		Adjusted	
	Odds Ratio (95% CI)	*p* value	Odds Ratio (95% CI)	*p* value
Age	1.10 (1.03 to 1.17)	0.004^*^	1.14 (1.04 to 1.24)	0.004^*^
Simple cyst (Y vs. N)	0.44 (0.12 to 1.59)	0.210	0.34 (0.05 to 2.44)	0.284
Mature cystic teratoma (Y vs. N)	0.79 (0.18 to 3.39)	0.746		
Endometrioma (Y vs. N)	2.44 (0.25 to 24.04)	0.446	0.37 (0.02 to 8.23)	0.532
Fibroma or fibrothecoma (Y vs. N)	1.05E9 (NA)	0.999		
Necrosis of ovary (Y vs. N)	9.32E8 (NA)	1.000		
Symptoms and signs				
Ovarian or pelvic mass (Y vs. N)	NA	NA		
Pelvic pain (Y vs. N)	0.00 (NA)	0.999		
Nausea and vomiting (Y vs. N)	0.25 (0.05 to 1.25)	0.092	0.02 (0.00 to 0.97)	0.048^*^
Peritoneal sign (Y vs. N)	9.69E8 (NA)	0.999		
WBC > 12,000 (Y vs. N)	3.18 (0.34 to 30.16)	0.313	42.03 (0.87 to 2,020.68)	0.059
Fever (Y vs. N)	9.32E8 (NA)	1.000		
Urinary symptoms (Y vs. N)	0.00 (NA)	0.999		
Diarrhea (Y vs. N)	0.54 (0.03 to 9.28)	0.670		

**Notes.**

Data are presented as Odds ratio (95% CI).

* *P*-value <  0.05 was considered statistically significant after test.

Y oophorectomy N no oophorectomy

## Discussion

In our series, 64.4% of patients were suspected to have adnexal torsion. In other reports, the incidence of suspected cases ranges from 18% to 62% before surgery (Cohen et al., 2001; Houry & Abbott, 2001; Oelsner et al., 2003). Suspicion of adnexal torsion is the key to early diagnosis (Cohen et al., 2001). Adnexal torsion should be suspected in patients with a history of an enlarged ovary or pelvic surgery complicated with abdominal pain (Houry & Abbott, 2001). Tubal ligation is the most frequent surgical history in 40% of cases with adnexal torsion (Houry & Abbott, 2001).

Table 7 compared the current study to the previous literatures. Abdominal pain accounted for the most presenting symptoms. Adnexal torsion was mostly happened at the Rt side. The tumor size was mostly between 5–10 cm. Laparoscopic surgery was the most surgical type. The proportion of conservative or radical surgery was variant in different studies. In our study, we found the older age is the risk factor for radical surgery. We speculated, for young patients, the conservation of ovary is important for their future fertility. Thus, conservative surgery is largely performed for young patients with ovarian torsion.

**Table 7:** Comparison of present study to the previous studies.

	Our study	Nair, Joy & Nayar (2014)	Vijayalakshmi et al. (2014)	Spinelli et al. (2013)	Vijayaraghavan (2004)
No. of patients	42	70	18	30	21
Age	12–82 yrs	11–91 yrs	25–72 yrs	2 months–18 yrs	7–69 yrs
Pregnancy	6.6%	2.9%			4.7%
Abdominal pain	95.2%	95.7%	77.8%	100%	100%
Nausea/vomiting	19%	65.7%	27.8%	56.7%	
Fever	2.3%	12.9%	5.6%	20%	
Leukocytosis	14.2%	44%		63.3%	
Doppler diagnosis of torsion		25.7%		63%	95.2%
Free fluids in pelvis		23.8%		26.7%	
Right side	51.1%	55.7%	50%	70%	
Left side	40.0%	42.9%	38.9%	30%	
Bilateral	2.2%	1.4%	11.1%		
Size <5 cm	11.1%				
Size 5∼10 cm	71.1%	71.4%	33.3%		
Size >10 cm	17.7%	11.4%	44.4%		
Mature cystic teratoma	23.8%	22.8%	16.7%	16.7%	28.5%
Mucinous cystadenoma		7.1%	33.3%	3.3%	9.5%
Serous cystadenoma		15.7%	16.7%		47.6%
Hemorrhagic necrosis	2.3%	30.4%			9.5%
Laparoscopy	64.2%	81.4%		40%	
Laparotomy	35.8%	18.6%		60%	
Conservative surgery	35.8%	54.3%		46.7%	
Radical surgery	64.2%	45.7%		53.3%	

The study regarding fertility after ovarian torsion surgery is lacking. One report showed patients with one ovary (mostly by oophorectomy due to ovarian torsion or other pathology) did not affect the reproductive capability (Lass, 1999). But they have a shorter reproductive life span due to less primordial follicles (Lass, 1999).

Objective findings are variable and are rarely significant in patients with adnexal torsion. A study of 179 patients with adnexal torsion showed leukocytosis and fever in 20.1% and 7.8% of the patients (Nair, Joy & Nayar, 2014). In our study, one patient had a fever (2.3%) and six patients had leukocytosis (14.2%).

The sensitivity of pelvic ultrasound for diagnosing adnexal torsion ranges from 40% to 75% (Mashiach et al., 2011). Twisted adnexal masses are often midline and located anterior to the uterus; other findings include a cystic, solid, or complex mass with or without pelvic free fluid and thickening of the wall with cystic hemorrhage (Mashiach et al., 2011). A previous study found adnexal tumors larger than five cm in 88.4% of patients with adnexal torsion (Nair, Joy & Nayar, 2014). The whirlpool sign over the vessel pedicle has been reported to be a sign of adnexal torsion (Vijayaraghavan, 2004). In our study, the absence of blood flow showed low sensitivity for diagnosing adnexal torsion.

In the present study, the most common pathology was a simple ovarian cyst, followed by teratoma. Ashwal et al. also showed that the most common pathology was a simple cyst (Ashwal et al., 2015). However, Lo et al. (2008) found that teratoma was the most common pathology.

Multiple tumor markers, such as AFP (alpha-fetoprotein), CEA (carcinoembryonic antigen), HCG (human chorionic gonadotropin), and CA125, have been used for diagnosing ovarian neoplasms. CA125 is used for diagnosing epithelial ovarian tumors; however, it shows poor sensitivity and specificity (Moss, Hollingworth & Reynolds, 2005). In addition, CA125 levels are elevated in other benign conditions such as adenomyosis and endometriosis (Ghaemmaghami, Karimi Zarchi & Hamedi, 2007). One case study reported the association between elevated CA125 levels and adnexal torsion (McCarthy et al., 2010). However, in an emergency setting, CA125 results cannot be rapidly obtained. Therefore, we did not routinely determine CA125 levels in our adnexal torsion cases.

C reactive protein (CRP) has been reported as a novel marker of adnexal torsion (Tobiume et al., 2011; Damigos, Johns & Ross, 2012; Bakacak et al., 2015; Bolli et al., 2017). In an animal experiment, after adnexal ischemia, the plasma CRP level was found to be elevated (Bakacak et al., 2015). In a previous study, a CRP level of <0.3 mg/dL was correlated with a favorable prognosis of ovarian conservation in adnexal torsion (Tobiume et al., 2011). However, in our study, we did not routinely evaluate CRP levels.

The main reason for surgical intervention in cases of adnexal torsion is lower abdominal pain and enlarged adnexal masses (Cohen et al., 2001). In our patients, the time from admission to surgery was 3 h, which was shorter than the time reported by Ashwal of 4.6 h (Ashwal et al., 2015).

In our study, no significant difference was observed in operative time between the laparoscopy and laparotomy groups. Lo et al. (2008) also found no significant difference in operative time between laparoscopy and laparotomy groups.

The only significant factor for choosing surgical route is tumor size (Lo et al., 2008). In our series, the mean diameter of adnexal tumors was 6.9 and 10.4 cm in the laparoscopy and laparotomy groups, respectively (*P* <  0.01). Significantly smaller ovarian cysts were noted in the laparoscopy group than in the laparotomy group.

Although adnexal torsion occurs in all age groups, it is most commonly observed in reproductive age groups (Tsafrir et al., 2012; Vijayalakshmi et al., 2014). In this study, most patients were postmenarchal, and eight women were postmenopausal.

In our study, younger patients received laparoscopy (33.6 vs. 41.0 years, *P* = 0.18). A previous study also showed no difference in age between the laparoscopy and laparotomy groups (30 vs. 32 years). Cosmetic reasons may affect the chosen surgical route such as minimal invasive laparoscopy (Lee et al., 2010). The other reason is that ovarian malignancy is more often suspected in older patients, who may choose laparotomy (Moorman et al., 2008).

Significantly shorter length of hospital stay was noted in the laparoscopy compared to the laparotomy group (4.0 vs 5.4 days, *p* < 0.01). This observation is consistent with the previous reports (Lo et al., 2008; Grammatikakis et al., 2015).

We have shown that laparoscopic detorsion and cystectomy are feasible in adults and pregnant women with adnexal torsion (Ding & Chen, 2005; Ding & Chang, 2016). Detorsion carries no risk of thrombosis (Ashwal et al., 2015; Huang, Hong & Ding, 2017). In recent years, detorsion without cystectomy or adnexectomy has become more prevalent (Ashwal et al., 2015).

In a previous study on a patient with a benign tumor, the potential for ovary conservation was found to be greater in adnexal torsion cases with a short period from the onset of abdominal pain to surgery (Tobiume et al., 2011). In our study, we performed detorsion with ovarian cystectomy irregardless of tumor size. Moreover, a benign ovarian pathology should be obtained during surgery (Jeon et al., 2017).

Previous studies have shown that ovarian morphology and function are restored to normal after detorsion (Galinier et al., 2009; Geimanaite & Trainavicius, 2013; Parelkar et al., 2014). Moreover, studies have suggested that conservative treatment of adnexal torsion is safe. After adnexal detorsion, menstruation and normal ovarian morphology were restored in all our premenopausal patients.

The recurrence rate after detorsion is remained unknown. One report revealed 63% and 8.7% recurrence rates in the twisted normal adnexa and twisted abnormal adnexa, respectively (Pansky et al., 2007). There are several methods could prevent recurrence including oophoropexy (Pansky et al., 2007) and oral contraceptives (Fee, Kanj & Hoefgen, 2017). However, both methods lack long term follow-up study.

## Conclusions

In conclusion, the early diagnosis and intervention of adnexal torsion are crucial. Acute onset of abdominal pain with a presenting adnexal tumor is the most common feature of adnexal torsion. Laparoscopic surgical group showed a small tumor size and a shorter hospital stay than laparotomy. Older age is the risk factor for radical surgery.

##  Supplemental Information

10.7717/peerj.5995/supp-1 Supplemental Information 1 Raw data

## Additional Information and Declarations

### Data Availability

The following information was supplied regarding data availability: The data is included in the results section and Supplemental Files.

## References

[ref-1] Ashwal E, Hiersch L, Krissi H, Eitan R, Less S, Wiznitzer A, Peled Y (2015). Characteristics and management of ovarian torsion in premenarchal compared with postmenarchal patients. Obstetrics and Gynecology.

[ref-2] Bakacak M, Köstü B, Ercan Ö, Bostancı MS, Kıran G, Aral M, Çıralık H, Serin S (2015). High-sensitivity C-reactive protein as a novel marker in early diagnosis of ovarian torsion: an experimental study. Archives of Gynecology and Obstetrics.

[ref-3] Bolli P, Schädelin S, Holland-Cunz S, Zimmermann P (2017). Ovarian torsion in children: development of a predictive score. Medicine.

[ref-4] Chang HC, Bhatt S, Dogra VS (2008). Pearls and pitfalls in diagnosis of ovarian torsion. Radiographics.

[ref-5] Cohen SB, Weisz B, Seidman DS, Mashiach S, Lidor AL, Goldenberg M (2001). Accuracy of the preoperative diagnosis in 100 emergency laparoscopies performed due to acute abdomen in nonpregnant women. The Journal of the American Association of Gynecologic Laparoscopists.

[ref-6] Damigos E, Johns J, Ross J (2012). An update on the diagnosis and management of ovarian torsion. Obstetrics and Gynecology.

[ref-7] Ding D-C, Chang Y-H (2016). Laparoendoscopic single-site surgical cystectomy of a twisted ovarian dermoid cyst during early pregnancy: a case report and literature review. Gynecology and Minimally Invasive Therapy.

[ref-8] Ding D-C, Chen SS (2005). Conservative laparoscopic management of ovarian teratoma torsion in a young woman. Journal of the Chinese Medical Association.

[ref-9] Fee EK, Kanj RV, Hoefgen HR (2017). Recurrent ovarian torsion in an adolescent after oophoropexy. Journal of Pediatric Surgery Case Reports.

[ref-10] Galinier P, Carfagna L, Delsol M, Ballouhey Q, Lemasson F, Le Mandat A, Moscovici J, Guitard J, Pienkowski C, Vaysse P (2009). Ovarian torsion. Management and ovarian prognosis: a report of 45 cases. Journal of Pediatric Surgery.

[ref-11] Geimanaite L, Trainavicius K (2013). Ovarian torsion in children: management and outcomes. Journal of Pediatric Surgery.

[ref-12] Ghaemmaghami F, Karimi Zarchi M, Hamedi B (2007). High levels of CA125 (over 1,000 IU/ml) in patients with gynecologic disease and no malignant conditions: three cases and literature review. Archives of Gynecology and Obstetrics.

[ref-13] Grammatikakis I, Trompoukis P, Zervoudis S, Mavrelos C, Economides P, Tziortzioti V, Evangelinakis N, Kassanos D (2015). Laparoscopic treatment of 1522 adnexal masses: an 8-year experience. Diagnostic and Therapeutic Endoscopy.

[ref-14] Houry D, Abbott JT (2001). Ovarian torsion: a fifteen-year review. Annals of Emergency Medicine.

[ref-15] Huang C, Hong M-K, Ding D-C (2017). A review of ovary torsion. Tzu-chi Medical Journal.

[ref-16] Huang B-S, Wang P-H (2011). Ovarian torsion during pregnancy. Taiwanese Journal of Obstetrics & Gynecology.

[ref-17] Jeon H, Ryu A, Seo H-G, Jang S-H (2017). Ovarian torsion of mixed epithelial tumor misdiagnosed as a malignancy in postmenopausal woman: a case report. Medicine.

[ref-18] Lass A (1999). The fertility potential of women with a single ovary. Human Reproduction Update.

[ref-19] Lee Y-Y, Kim T-J, Kim C-J, Park HS, Choi CH, Lee J-W, Lee J-H, Bae D-S, Kim B-G (2010). Single port access laparoscopic adnexal surgery versus conventional laparoscopic adnexal surgery: a comparison of peri-operative outcomes. European Journal of Obstetrics, Gynecology, and Reproductive Biology.

[ref-20] Lo L-M, Chang S-D, Horng S-G, Yang T-Y, Lee C-L, Liang C-C (2008). Laparoscopy versus laparotomy for surgical intervention of ovarian torsion. The Journal of Obstetrics and Gynaecology Research.

[ref-21] Mashiach R, Melamed N, Gilad N, Ben-Shitrit G, Meizner I (2011). Sonographic diagnosis of ovarian torsion: accuracy and predictive factors. Journal of Ultrasound in Medicine.

[ref-22] McCarthy JD, Erickson KM, Smith YR, Quint EH (2010). Premenarchal ovarian torsion and elevated CA-125. Journal of Pediatric and Adolescent Gynecology.

[ref-23] Moorman PG, Calingaert B, Palmieri RT, Iversen ES, Bentley RC, Halabi S, Berchuck A, Schildkraut JM (2008). Hormonal risk factors for ovarian cancer in premenopausal and postmenopausal women. American Journal of Epidemiology.

[ref-24] Moss EL, Hollingworth J, Reynolds TM (2005). The role of CA125 in clinical practice. Journal of Clinical Pathology.

[ref-25] Nair S, Joy S, Nayar J (2014). Five year retrospective case series of adnexal torsion. Journal of Clinical and Diagnostic Research.

[ref-26] Oelsner G, Cohen SB, Soriano D, Admon D, Mashiach S, Carp H (2003). Minimal surgery for the twisted ischaemic adnexa can preserve ovarian function. Human Reproduction.

[ref-27] Pansky M, Smorgick N, Herman A, Schneider D, Halperin R (2007). Torsion of normal adnexa in postmenarchal women and risk of recurrence. Obstetrics and Gynecology.

[ref-28] Parelkar SV, Mundada D, Sanghvi BV, Joshi PB, Oak SN, Kapadnis SP, Shetty S, Athawale H, Multani P (2014). Should the ovary always be conserved in torsion? A tertiary care institute experience. Journal of Pediatric Surgery.

[ref-29] Spinelli C, Buti I, Pucci V, Liserre J, Alberti E, Nencini L, Alessandra M, Lo Piccolo R, Messineo A (2013). Adnexal torsion in children and adolescents: new trends to conservative surgical approach—our experience and review of literature. Gynecological Endocrinology.

[ref-30] Tobiume T, Shiota M, Umemoto M, Kotani Y, Hoshiai H (2011). Predictive factors for ovarian necrosis in torsion of ovarian tumor. The Tohoku Journal of Experimental Medicine.

[ref-31] Tsafrir Z, Hasson J, Levin I, Solomon E, Lessing JB, Azem F (2012). Adnexal torsion: cystectomy and ovarian fixation are equally important in preventing recurrence. European Journal of Obstetrics, Gynecology, and Reproductive Biology.

[ref-32] Vijayalakshmi K, Reddy GMM, Subbiah VN, Sathiya S, Arjun B (2014). Clinico-pathological profile of adnexal torsion cases: a retrospective analysis from a tertiary care teaching hospital. Journal of Clinical and Diagnostic Research.

[ref-33] Vijayaraghavan SB (2004). Sonographic whirlpool sign in ovarian torsion. Journal of Ultrasound in Medicine.

